# An assessment of the nutritional status of ART receiving HIV-orphaned and vulnerable children in South-West Nigeria

**DOI:** 10.1016/j.heliyon.2019.e02925

**Published:** 2019-12-09

**Authors:** Adeniyi Francis Fagbamigbe, Ayo Stephen Adebowale, IkeOluwapo Ajayi

**Affiliations:** Department of Epidemiology and Medical Statistics, College of Medicine, University of Ibadan, Nigeria

**Keywords:** Public health, Epidemiology, HIV/AIDS, BMI-for-age, ART, Nutrition, Weight-for-age, Height-for-age

## Abstract

**Introduction:**

Good nutritional status is pertinent to the optimal outcome of effective ART among children. Against this backdrop, the objective of the current study is to assess the nutritional indices of children receiving ART in South-West Nigeria.

**Methods:**

The study was cross-sectional in design. We randomly selected three urban and six rural ART sites from the ones offering ART services in Oyo state. All consented children receiving ART treatments in the aforementioned sites participated in the study. A total of 390 HIV-positive children and adolescents aged 6–18 years were interviewed using a semi-structured interviewer-administered questionnaire. Children were assessed and growth curves were constructed using the 2007 World Health Organisation (WHO) growth reference standard for children as well as adolescents. Data were presented using descriptive statistics.

**Results:**

About 52% of the children are male, 136 (34.9%) have lost at least one parent, 52 (13.3%) have lost either parent to HIV/AIDS. Among the males, 19%, 27%, and 27% were underweight, stunted and thin, respectively when compared with 17%, 23% and 23%, respectively, among females. The male and female weight-for-age average z-score were (-0.98 vs -1.04), height-for-age (-1.12 vs -1.07), and BMI-for-age (-1.19 vs -1.18). Irrespective of age, sex, parental survival, and residence, weight-for-age and BMI-for-age analysis revealed substantial underweight, with the worst outcomes being among those orphaned by HIV/AIDS.

**Conclusion:**

All nutritional indices considered in this study fell short of the WHO standard. HIV positive children in the ART sites included in this study are faced with a high burden of undernourishment despite been placed on daily ART regimens. In addition to efficient ART, interventions to ameliorate poor nutritional status is needed.

## 1 Introduction

Globally, about 37 million people were living with HIV in 2017, of which 1.8 million were accounted for by children aged 15 years or younger (UNAIDS, 2018b). In that same year, the western and central Africa sub-regions hosted for 6.1 million people living with HIV (PLHIV). Nigeria, the most populous African country, had 1.9 million PLHIV as of 2018, with an HIV prevalence of 1.4% (Federal Government of Nigeria, 2019; UNAIDS, 2018b), one of the highest prevalence among neighbouring western Africa countries (USAID, 2012). In that context, Antiretroviral Therapy (ART) has been introduced worldwide in order to maintain PLHIV. The tremendous advantages of ART adherence are replete in extant literature (Lodha and Kabra, 2015; National Agency for the Control of AIDS (NACA), 2015; Sunguya et al., 2011; Swetha et al., 2015). An estimated 21.7 million people were receiving ART at the end of 2017 all over the world (UNAIDS, 2018a). However, only 941,000 Children Living with HIV (CLHIV) had access to ART, which translated to only 52% coverage in comparison with 59% coverage observed in the adult population (UNAIDS, 2018a). Although the incidence and prevalence of HIV among children, diagnosis, and treatment of CLHIV remain a challenge (UNAIDS, 2018a), HIV treatment services are essentially designed for adults with limited consideration to meet the specific needs of younger individuals (Govindasamy et al., 2015).

Since the Federal Government of Nigeria (FGN) introduced the free ART treatment policy in 2006, ART coverage has increased tremendously (National Agency for the Control of AIDS (NACA), 2015). However, as of 2014, only 45% of the 1.6 million persons in need of ART were under care (National Agency for the Control of AIDS (NACA), 2015). Despite the commendable achievements of ART in Nigeria, gaps such as inadequate access to ART among children, weak referral systems between community care centres and facilities providing ART services, poor procurement and supply chain management, paucity in the number of competent human resources; poor coordination between directly observed therapy (DOT) sites andART sites, variation in the quality of care and treatment services, and low level of collaboration/engagement with the private sector have been identified (National Agency for the Control of AIDS (NACA), 2015).

About 6% of Nigerian children under the age of-18 years are reported to have one or both parents deceased; to that end, evidence showed that 9% of children are orphans or vulnerable due to illnesses among adult household members (National Agency for the Control of AIDS (NACA), 2015). Only about 5% of Orphaned and Vulnerable Children (OVC) ever receive any type of medical, emotional, social, material or school-related assistance (National Population Commission (Nigeria) and ICF International., 2014). Additionally, it has been reported that HIV/AIDS is a major cause of parental deaths among OVC living with HIV/AIDS, particularly in households where both parents have died (National Agency for the Control of AIDS (NACA), 2015). In addition, the loss of parents may lead to social and economic vulnerability of their surviving children (Sunguya et al., 2011; Turck et al., 2013).

Nutrition plays a vital role in disease infection cycles (Lodha and Kabra, 2015). Undernutrition is one of the top killers among children given that it accounts for over 35% of preventable deaths among children (WHO, 2019). Poor nutrition is one of the identified challenges of PLHIV because it accelerates the progression of HIV into full AIDS (Lodha and Kabra, 2015; WHO, 2019). A close and dual-way relationship has been established between HIV and malnutrition (Lodha and Kabra, 2015). While HIV compromises nutritional status, on the one hand, poor nutrition weakens body immunity on the other hand. More specifically, malnutrition makes heavy energy demands on children's bodies and more crucially, their immune system (Lodha and Kabra, 2015). Nine out of every ten CLHIV usually are confronted with delayed growth. A person living with HIV and those who are also malnourished are at heightened risk of opportunistic infections (Ndondoki et al., 2011). These opportunistic infections, in turn, exacerbate poor nutrition primarily due to the weakened immune systems of PLHIV. Opportunistic infections are often recurrent and have tendencies of spreading at a fast pace. Most importantly, malnutrition can worsen the survival chances of CLHIV and often lead to the slow rate of viral load suppression, poor response to treatments, and a quick progression of HIV to full-blown AIDS (Sunguya et al., 2011).

Children with weight-for-height <-2 z-score from the reference population median are considered to be thin (wasted) or acutely malnourished, while those who have height-for-age <-2 z-score are referred to as stunted (chronic malnutrition). Children whose weight-for-age is <-2 z-score based on the reference population median are adjudged underweight. In addition, BMI-for-age is a composite score of height-for-age and weight-for-height that measures thinness among school-age children (National Population Commission (Nigeria) and ICF International., 2014; Valente et al., 2016). As of the last general national survey data in Nigeria, 37% of under-five children were stunted, 18% were wasted, and 29% were underweight as compared to 27%, 10% and 18%, respectively in the Oyo state, respectively (National Population Commission (Nigeria) and ICF International., 2014).

Despite the extensive provision of ART, malnutrition among CLHIV remains both clinical and public health challenge (Fekadu et al., 2015; Sunguya et al., 2011). Most studies on malnutrition had hitherto been restricted to the general population and ART-naive CLHIV. This, in turn, has made generalization of such findings unreasonable to CLHIV on ART (Sunguya et al., 2011). Therefore, timely/efficient identification and intervention to halt malnutrition among HIV-positive children can accelerate quick recovery from infection, enhance the ability of body antigens to prevent further infection, and lead to a likely reduction in the rate of progression from HIV to full-blown AIDS. Accordingly, the objective of this study is to examine the nutritional status of children receiving ART in Nigeria's Oyo State.

## 2 Methods

The data for this study were collected from CLHIV attending ART sites of the Presidents Emergency Plan for AIDS Relief (PEPFAR) and Oyo state Agency for the Control of AIDS (OYSACA) in Oyo State, Nigeria. Data collection exercise lasted for a period of four months i.e. from March to June 2015. As one of the 36 states in Nigeria, Oyo is located in the South-West geopolitical zone. The state is home to over 7 million people in 2013 based on a 2006 projection (National Population Commission, 2013) using an estimated annual growth rate of 3.2% per annum (National Population Commission (Nigeria) and ICF International., 2014; National Population Commission, 2013; WHO, 2014).

### 2.1 Study design and sampling procedure

The study was cross-sectional in design. We randomly selected three urban and six rural ART sites among the sites run by the PEPFAR and OYSACA in the state of Oyo. Due to the limited number of children aged 6–18 years visiting the sites, we recruited all children receiving ART treatments in the sites. A total of 390 HIV-positive children aged 6–18 years were interviewed using a semi-structured interviewer-administered questionnaire. The questionnaire consisted of questions relating to the children's demographics, family history, OVC support system, and other relevant issues on children living with HIV/AIDS. Furthermore, other family background information and survival status of parents were collected as well, while anthropometric measurements were taken using internationally approved procedures and as per their usage in previous studies (Maiti et al., 2012; WHO, 2009a, 2009b; Yallamraju et al., 2014). In order to minimize bias and errors, the heights of the studied children were measured in conjunction with the weight measurement. In particular, we used validated calibrated balance beam scales. The scales were placed on a hard floor surface and balanced at zero weights before taking the measurements. The height rulers were taped vertically to a hard, flat surface, without moulding with the base at the floor level. We also used the carpenter's level to ensure vertical placement of the ruler on a hard floor surface.

### 2.2 Data analysis

Descriptive statistics were used to describe the study participants' and nutritional characteristics. To that end, we conducted a test of equality of proportions using Stata version 12.0 at 5% significant level. The World Health Organisation (WHO) “anthro plus”, an application dedicated to the evaluation of the growth of children aged 6–19 years vis-à-vis gender and age, was used to construct the children's growth curves alluded to in this study. The application was based on the 2007 WHO growth reference standard for school-aged children and adolescents aged 6–19 years. The outcomes were evaluated across the participants' background characteristics. More specifically, we computed the weight-for-age (WFA), height (length)-for-age (HFA), and BMI-for-age (BFA).

We assessed the nutritional status of the children and correlated it with the parental living status along with other characteristics, including social-economic status, parents' educational status, etc. In addition, we constructed the participants’ growth curves using the 2007 WHO growth reference standard for both school-aged children and adolescents, thus disaggregating the nutritional status by gender, place of residence, and parental survival. The need for parental status is contingent on the fact that some of the children had lost one or both parents to HIV/AIDS.

Typically, the charts have five curves, with the middle curve being the median. Other lines on the curve, referred to as z-score lines, indicate the distance from the median curve. Points that are far from the median, such as a +3, +2, -2, or -3, typically indicate the growth problem. For the HFA, children are considered to be stunted when their HFA falls below the -2 z-score line. Similarly, children are considered to be underweight if their WFA falls below the -2 z-score line. A point at -3 z-score lines of the WFA indicates severe underweight and undernourishment, thus suggesting the need for special care. Meanwhile, severe underweight may be a result of stunting, wasting, or both (De Onis et al., 2007; Turck et al., 2013; WHO, 2010).

The BFA z-score above +1SD suggest overweight, values higher than +2SD implies obesity, while a score below -2SD and -3SD indicates thinness and severe thinness, respectively. While the HFA and BFA are recommended/computed for all children as well as adolescents aged 6–19 years, WFA is specifically reserved for the children aged 6–9 years. The usage of growth charts in showing graphical anthropometry characteristics in a population, including the calculated z-score for body mass index (BMI), height, and weight, has been described as “critical to health-care providers assessing patterns of growth, body shape, and size” (McDonald et al., 2013; Rodd et al., 2014).

## 3 Result

Of the 390 children included in the analysis, about 52% of the children are male, 35% were aged 6–9 years, and 44% aged 10–14 years, whereas 21% were aged 15–18 years (Table 1). About 80% of the children are urban dwellers, and their fathers mostly had primary (36%) and secondary (28%) education as compared to 44% and 32%, respectively among their mothers. Over a quarter (103 (27%)) have lost one parent, 27 (7%) have lost both parents, while 58% had parents who currently live together. Of the 130 children who reported to have lost at least one parent, 21% lost only mothers while 12% only fathers to HIV/AIDS. In a similar vein, only 7% had lost their both parents to HIV/AIDS.

**Table 1:** Socio-demographic characteristics of the children.

Characteristics	n	%
**Age in years**		
5–9	136	34.9
10–14	172	44.1
15–18	82	21.0

**Sex**		

Male	203	52.0
Female	187	48.0

**Residence**		
Urban	311	79.7
Rural	79	20.3

**Parents Living status**		
Parents Living together	225	57.8
Divorced/separated	34	8.7
One is dead	103	26.5
Both dead	27	6.9

**Death due to HIV/AIDS**		
Maternal to AIDS	29	21.2
Paternal to AIDS	16	11.7
Both to AIDS	7	5.1
Neither to AIDS/Unknown	85	62.0

**Father Education**		
No formal education	13	3.3
Primary	141	36.3
Secondary	108	27.8
Tertiary	86	22.1
Unknown	41	10.5

**Father occupation**		
Farming	32	8.3
Trading/businessman	163	42.0
Civil servant	51	13.1
Private sector	40	10.3
Others	102	26.3

**Mother Education**		
No formal education	22	5.7
Primary	169	43.9
Secondary	120	31.8
Tertiary	63	16.4
Unknown	11	2.8

**Mother occupation**		
Farming	25	6.4
Trading/businessman	253	65.2
Civil servant	44	11.34
Private sector	8	2.1
Others	58	14.7
Total	390	100

As illustrated in Table 2, the analysis of WFA among children aged 6–9 years revealed that 5.7% of male children had less than -3SD, as a result of which they were severely underweight, as compared to 9.1% among female children. Also, 18.6% among males and 16.7% among females reported WFA z-score below -2SD and thus were deemed underweight, whereas others had acceptable WFA. The analysis of HFA illustrates that 9.4% of males and 7.0% of females had z-score below -3SD (severely stunted), 26.7% males and 22.5% of females had z-score below -2SD (stunted). Additionally, the BFA analysis pointed out that 12.9% of the 203 males and 8.7% of the 187 females have z-score less than -3SD, which indicates severe thinness, while 26.9% and 23.4% of males and females respectively had less than -2SD z-score, an indicator of thinness among children. The proportions of children who were overweight, stunted, and thin were found to be higher among males. The average z-score for WFA, HFA, and BFA among the males and females was -0.98, -1.12 and -1.19, as well as -1.04, -1.07 and -1.18, respectively. In addition, all the scores were all below zero (the median), which suggests malnourishment among the studied children. The HFA was found to be lower among both male and female children aged 12 years or younger as compared to teenagers. Also, the BFA seemed to be lower among female teenagers in comparison to female children. For instance, 52.2% of male teenagers compared with 10.5% among children aged 7 years were found to be stunted.

**Table 2:** Distribution of z-score for Weight-for-age. height-for-age and BMI-for-age by sex.

Age in years		Weight-for-age∗∗ (%)			Length/height-for-age∗ (%)			BMI-for-age∗ (%)		
		<-3SD	<-2SD	Median	<-3SD	<-2SD	Median	<-3SD	<-2SD	Median
Male	Number									
6	6	0.00	0.00	-1.31	0.00	0.00	-0.12	16.7	50.00	-2.03
7	19	5.3	15.8	-0.58	0.0	10.5	-0.04	10.5	42.1	-0.95
8	18	11.1	16.7	-1.08	5.6	16.7	-0.78	5.9	5.9	-0.62
9	27	3.7	25.9	-1.13	7.4	18.5	-0.77	11.1	22.2	-1.06
10	21				9.5	23.8	-0.63	14.3	33.3	-1.35
11	19				15.8	21.1	-1.40	10.5	15.8	-0.47
12	22				4.5	18.2	-0.99	9.1	13.6	-0.62
13	12				8.3	41.7	-1.54	0.0	16.7	-1.16
14	17				11.8	47.1	-1.70	17.6	29.4	-1.61
15	23				17.4	52.2	-1.96	21.7	39.1	-1.81
16	5				0.0	20.0	-1.61	20.0	40.0	-2.29
17	8				12.5	25.0	-2.05	25.0	50.0	-1.97
18	5				40.0	60.0	-2.35	20.0	20.0	-1.93
Total	203	5.7	18.6	-0.98	9.4	26.7	-1.12	12.9	26.9	-1.19

Female										

6	7	0.0	28.6	-1.11	0.0	0.0	0.02	0.0	42.9	-1.63
7	22	9.1	18.2	-0.81	0.0	4.5	0.03	18.2	27.3	-1.29
8	22	4.5	4.5	-0.91	0.0	9.1	-0.38	4.8	19.0	-0.85
9	15	20.0	26.7	-1.56	20.0	26.7	-1.52	13.3	20.0	-0.87
10	20				10.0	30.0	-1.25	5.0	20.0	-1.1
11	8				25.0	37.5	-1.7	0.0	0.0	-0.27
12	24				4.2	29.2	-1.43	8.7	30.4	-1.51
13	16				0.0	25.0	-1.39	12.5	31.3	-1.77
14	12				8.3	41.7	-1.97	9.1	27.3	-1.43
15	16				18.8	18.8	-1.46	6.3	25.0	-1.24
16	13				0.0	15.4	-0.77	0.0	15.4	-0.99
17	7				0.0	42.9	-1.37	14.3	14.3	-0.86
18	5				20.0	40.0	-1.64	20.0	20.0	-0.97
Total	187	9.1	16.7	-1.04	7.0	22.5	-1.07	8.7	23.4	-1.18

∗ ) Values are based on WHO standards (birth to 60 months) and WHO reference 2007 (61 months–19 years). Percentages below median based on weight-dependent indicators are defined as <-3 SD and <-2 SD.

∗∗ ) Weight-for-age reference data are not available beyond age 10 because this indicator does not distinguish between height and body mass in an age period where many children are experiencing the pubertal growth spurt and may appear as having excess weight (by weight-for-age) when in fact they are just tall.

**Figure 1:**
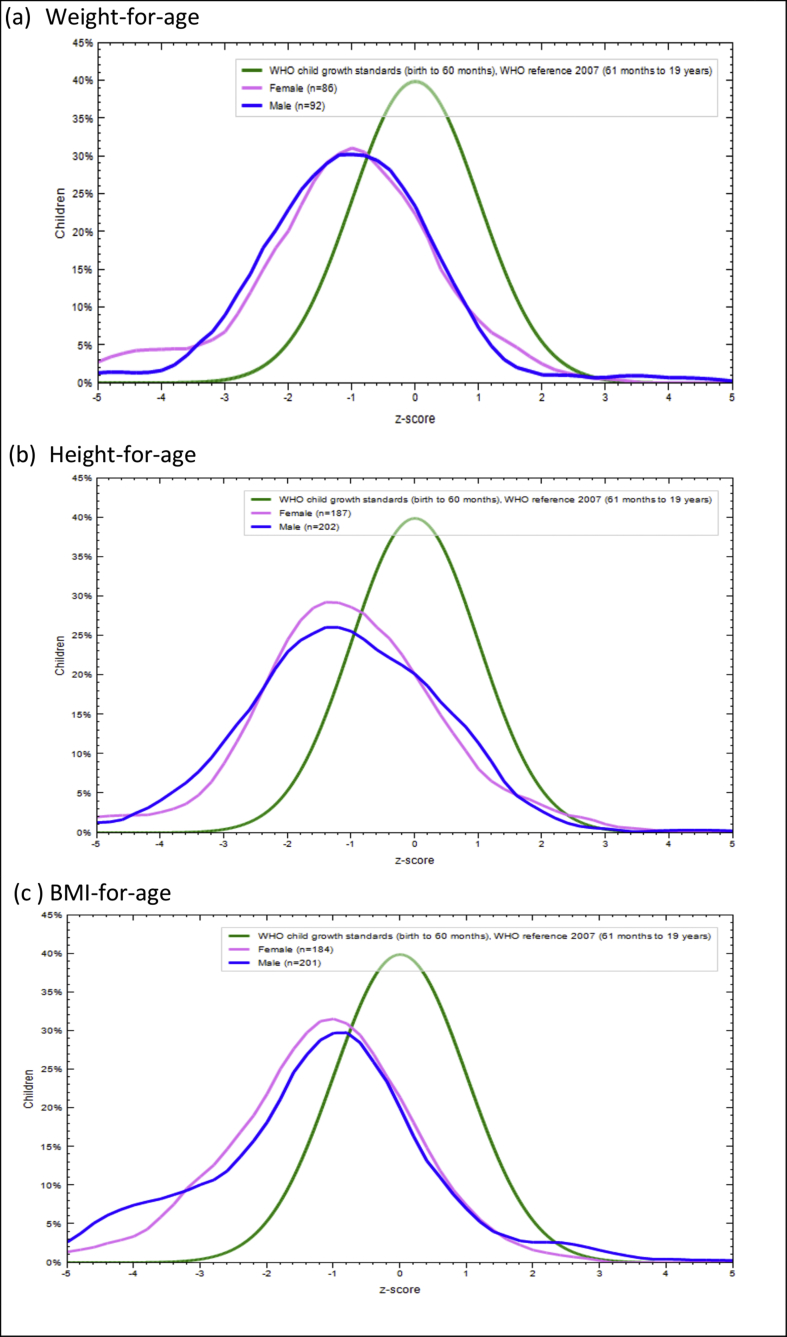
Distribution of Z-score of participants' Weight-for-age, height-for-age and BMI-for-age by sex.

**Figure 2:**
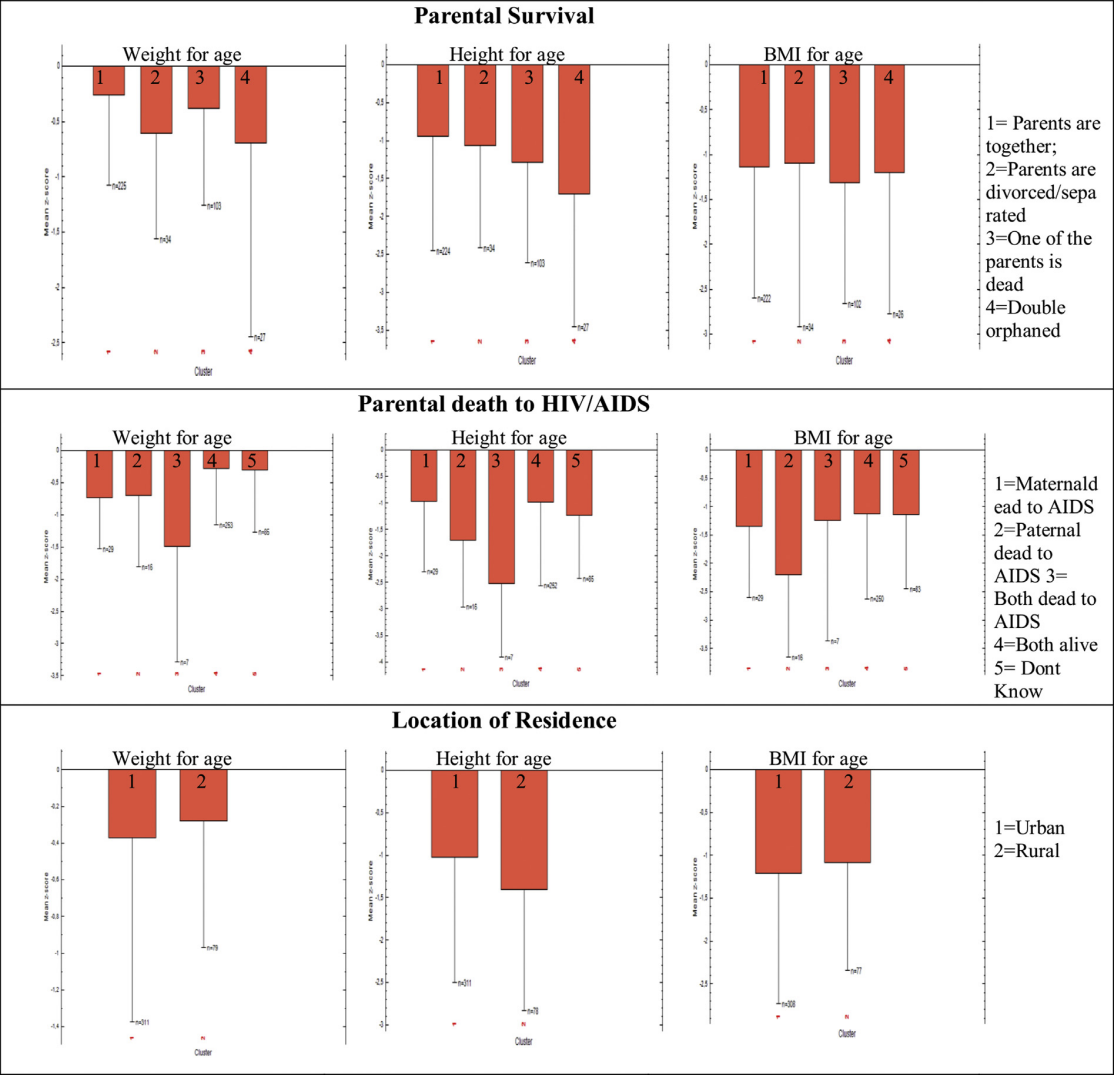
The mean z-score for WFA, HAG, and BFA of the children.

Figure 1 shows the WFA, HFA and BFA plots. It illustrates the evidence of underweight, stunting, and thinness among the children. The curves were all to the left side of, shorter, and wider than the median of the WHO child growth standard curve. The male and female weight-for-age average z-score were (-0.98 vs -1.04), height-for-age (-1.12 vs -1.07), and BMI-for-age (-1.19 vs -1.18). However, as shown by the figure, the prevalence of underweight among the CLHIV is more severe than stunting. Moreover, all the growth and nutrition indicators appear to be less severe among female CLHIV than their male counterparts. The median z-score for WFA, HFA, and BFA of the participants by selected characteristics are illustrated in Figure 2.

On parental survival, it is evident that underweight is more severe among double orphaned children followed by those whose parents were divorced or separated. Stunting is also found to be more pronounced among double orphaned children, those who had lost only one parent, or from separated parents - in that order. No major differences were found in thinness across the living status of the children's parents. With regard to the causes of deaths of the parents, underweight and stunting were predominantly higher among double orphaned children owing to HIV/AIDS. Furthermore, underweight and thinness were higher among urban CLHIV as compared to those living in rural areas, but the reverse is the case in stunting.

Among the males, 18.6% of those aged 6–9 years were found to be underweight, 26.1% in urban areas, and 20.3% in rural areas (p < 0.05). On father's educational attainment, 50.0% among children whose fathers had no formal education, 25.0% had primary education, 24.0% secondary had education, and 8.3% has tertiary education (p < 0.05). Underweight was found among 13.0% of male children whose both parents were living together, 33.3% among those whose parents were separated or divorced, 30.0% among children who had lost only one parent, and 44.4% among double orphaned children (p < 0.05). On the causes of parental death, underweight was found among 20.0% of those that lost only mothers to HIV/AIDS, 25.0% for those with paternal deaths, 50.0% among those that lost both parents to HIV/AIDS, and 37.5% among those that did not know what caused the demise of their parents (p < 0.05) as presented in Table 3.

**Table 3:** Distribution of WFA. HFA. and BFA among male children by selected characteristics.

Characteristics	^+^Weight for age (%)		Height for age (%)		BMI for age (%)		Number
	^a^Underweight	^b^Severe underweight	^a^Stunting	^b^Severe stunting	^a^Thinness	^b^Severe Thinness	
**Age**							
6–9 years	18.6	5.7	*14.3	4.3	*27.1	11.4	70
10–14 years	na	nc	28.3	9.8	21.7	10.9	92
15–18 years	nc	nc	43.9	17.1	39.0	22.0	41

**Residence**							
Urban	20.3	4.4	*24.5	9.4	28.9	15.1	159
Rural	26.1	8.7	34.1	9.1	20.5	6.8	44

**Father Education**							
No Schooling	50.0	0.0	-28.6	0.0	14.3	14.3	7
Primary	25.0	3.1	27.9	11.8	23.5	10.3	68
Secondary	24.0	4.0	23.2	3.6	33.9	17.9	56
Tertiary	8.3	0.0	26.5	8.2	26.5	8.2	49

**Mother Education**							
No schooling	33.3	0.0	*44.4	*11.1	*22.2	0.0	9
Primary	29.7	8.1	27.9	10.5	26.8	15.1	86
Secondary	17.2	6.9	26.7	11.7	31.7	15.0	60
Tertiary	10.5	0.0	21.6	5.4	24.3	10.8	37

**Parental Survival**							
Parents are living together	13.0	5.6	*27.1	8.5	*28.0	11.0	118
Parents are divorced/separa	33.3	0.0	15.8	10.5	21.1	21.1	19
One parent is dead	30.0	0.0	23.9	6.5	23.9	13.0	46
Both parents are dead	44.4	22.2	42.1	21.1	36.8	21.1	19

**Parent lost to HIV/AIDS**							
Maternal	20.0	0.0	*21.4	14.3	*28.6	7.1	14
Paternal	25.0	0.0	22.2	0.0	44.4	22.2	9
Both parents	50.0	50.0	60.0	20.0	40.0	40.0	5
Don't know	37.5	6.3	27.9	9.3	25.6	16.3	43

**Self-assessment of health status**							
Good	20.0	3.3	*26.7	9.6	*29.6	12.6	135
Not so good	27.6	10.3	27.9	8.2	19.7	14.8	61
Bad	0.0	0.0	16.7	16.7	50.0	16.7	6
Total	18.6	5.7	26.6	9.4	27.2	13.4	203

^b^included in ^a +^Computed among those age 6–9 years *Significant at 5% nc Not Computed (Not provided for in Anthro plus software).

Regarding stunting among males, it was found that 27.1% of male children whose both parents were together, 15.8% among those whose parents were separated or divorced, 23.9% among children who had lost only one of the parents, and 42.1% among those that had lost both parents (p < 0.05). On the causes of parental death, stunting was found among 21.4% of those that lost only mothers to HIV/AIDS, 22.2% among those that lost fathers to HIV/AIDS, 60.0% among double orphaned children to HIV/AIDS, and 27.9% among those that did not know what led to the demise of their parents (p < 0.05). Also, thinness was observed among 28.6% of those that lost only mothers to HIV/AIDS, 44.4% among those that lost fathers to HIV/AIDS, 40.0% among those that lost both parents to HIV/AIDS, and 25.6% among those that did not know the reason behind the death of their parents. The analysis of variance on the z-score by the selected characteristics of male children showed that HFA differed significantly by the ages of male children, while significant differences were also observed in the BFA by ages and self-assessment of the children's health status.

Among the female participants, 16.7% of those aged 6–9 years were found to be underweight, 21.1% in urban areas, and 28.6% in rural areas were also underweight (p < 0.05). Underweight was found among 10.9% of female children whose both parents were living together, 33.3% apiece among those whose parents were separated or divorced, as well as among children who had lost only one of the parents and 75.0% among those that had lost both parents (p < 0.05). In terms of the causes of parental death, overweight was found among 11.1% of those that had lost only mothers to HIV/AIDS, 66.7% for fathers, the only two children that had lost both parents to HIV/AIDS were found to be underweight and 33.3% among those that did not know the cause of the death of their parents (p < 0.05) as shown in Table 4.

**Table 4:** Distribution of WFA. HFA. and BFA among female children by selected characteristics.

Characteristics	^+^Weight for age (%)		Height for age (%)		BMI for age (%)		Number
	^a^Underweight	^b^Severe underweight	^a^Stunting	^b^Severe Stunting	^a^Thinness	^b^Severe Thinness	
**Age**							
6–9 years	16.7	9.1	*10.6	4.6	*25.8	12.1	66
10–14 years	nc	nc	31.3	7.5	26.3	10.0	80
15–18 years	nc	nc	24.4	9.8	19.5	7.3	41

**Residence**							
Urban	20.3	10.1	21.1	6.6	*27.0	11.2	152
Rural	28.6	14.3	28.6	8.6	14.3	5.7	35

**Father Education**							
No Schooling	100.0	100.0	*66.7	16.7	33.3	33.3	6
Primary	18.0	10.3	26.0	11.0	24.7	8.2	73
Secondary	20.0	8.0	23.1	7.7	23.1	13.5	52
Tertiary	16.7	11.1	8.1	0.0	21.6	5.4	37

**Mother Education**							
No schooling	40.0	40.0	*38.5	23.1	*7.7	7.7	13
Primary	20.5	10.3	26.5	8.4	27.7	9.6	83
Secondary	17.9	7.1	15.0	5.0	25.0	10.0	60
Tertiary	15.4	7.7	19.2	0.0	26.9	15.4	26

**Parental Survival**							
Parents are living together	*10.9	5.5	*18.7	6.5	23.4	9.4	107
Parents are divorced/separa	33.3	16.7	13.3	0.0	33.3	13.3	15
One parent dead is dead	33.3	19.1	28.1	8.8	28.1	12.3	57
Both parents are dead	75.0	25.0	50.0	12.5	0.0	0.0	8

**Parent lost to HIV/AIDS**							
Maternal	*11.1	0.0	*6.7	0.0	26.7	6.7	15
Paternal	66.7	66.7	57.2	14.3	57.2	42.9	7
Double orphaned	100.0	50.0	100.0	50.0	0.0	0.0	2
Don't know	33.3	8.3	26.2	2.4	19.0	7.1	42

**Self-assessment of health status**							
Good	16.4	10.9	*21.0	5.0	*27.7	10.1	119
Not so good	31.0	10.3	25.4	10.2	20.3	11.9	59
Bad	0.0	0.0	40.0	20.0	20.0	0.0	5
Total	16.7	9.1	22.5	7.0	23.4	8.7	187

^b^included in ^a^^+^Computed among those age 6–9 years *Significant at 5% nc Not Computed (Not provided for in Anthro plus software).

About 18.7% of female children whose both parents were living together stunted, 13.3% among those whose parents were separated or divorced, 28.1% among children who had lost only one of the parents, and 50.0% among those that had lost both parents. On the causes of parental death, stunting was found among 6.7% of those that lost only mothers to HIV/AIDS, 57.2% among those that lost fathers to HIV/AIDS, the two female children that lost both parents to HIV/AIDS were stunted and 26.2% among those that did not know what caused their parents' demise. Furthermore, 26.7% of those that lost only mothers to HIV/AIDS were stunted, 57.2% among those that lost fathers to HIV/AIDS, and 19.0% among others. The analysis of variance on the female children's z-score by the selected characteristics showed significant differences in the WFA of female children by parental marital and death status. Meanwhile, the HFA z-score was found to differ significantly by children age, fathers' and mothers' educational attainments, and parental survival of female children. Additionally, significant differences were found in the BFA z-score by whether the parents died as a result of HIV/AIDS.

## 4 Discussion

The current study assessed the nutritional status of CLHIV and receiving ART in Oyo State, situated in south-west Nigeria. We found that the nutritional indices considered among the children all fell short of the WHO standard. The levels of stunting, thinness and underweight among these children are high at about 1 in every 5 for each. The severity of underweight was found to be higher among children who had lost both parents, closely followed by those who live with one of their parents. The levels of thinness, stunting, and underweight found in the current study were found to be higher than those found in the general population across Nigeria (National Population Commission (Nigeria) and ICF International., 2014), but lower than 36.6% stunting and 22.1% underweight found among HIV positive children that were receiving ART in Ethiopia (Sunguya et al., 2011). Our results also demonstrated that children who lost one or both parents owing to HIV/AIDS were more predisposed to underweight, thinness, and stunting. Similar outcomes have been reported in the literature (Hailemariam et al., 2013; Moolasart et al., 2017; Nsagha, 2012; Poda et al., 2017; Sunguya et al., 2011). Inability to collect data on viral loads among the studied children is one of the limitations of this study, given that this could have made the provision to assess whether these children had viral suppression or not.

We found a higher prevalence of malnutrition among orphaned CLHIV. This is particularly exacerbated by the fact that at least 25% of the studied children have lost one parent and about 10% have lost both parents to either HIV/AIDS or other causes. This finding is similar to the outcome of an earlier study which reported that 25% of HIV positive children in Cameroon are actually orphaned (Nsagha, 2012). In 2017, as many as 1.8 million children globally were orphaned by AIDS (AVERT, 2019). Inexorably, this pattern has an implication on the level of household support being received by the children. Support in health care and food provision to children by foster parents may not be comparable to that which is provided by biological parents. This could be attributed to the fact that individual surrogate families are already over-burdened with the provision of daily needs for their own children (Umeora et al., 2013), and that they might view foster children, particularly those who live with HIV/AIDS, as an additional burden and “risk”.

Severe underweight was found among some of the CLHIV aged 6–9 years, but the level was higher among male than their female counterparts. All the growth and nutrition indices assessed in the present study, excluding BFA, seem to be less severe among female children as compared to their male counterparts. Approximately one in five and one-sixth of females and males, respectively, were underweight, an indication that the likelihood of underweight was slightly higher among females than males. However, our finding is at variance with a report by Moolasart et al. which did not find any significant difference in malnutrition between males and females CLHIV (Moolasart et al., 2017). Regardless of the age of the children, we found that the severity of underweight and thinness was higher among urban CLHIV than their rural counterparts, but the reverse was observed when it comes to stunting. The study shows that the proportions of male children who were overweight, stunting, or thin were higher than in females. Meanwhile, the height-for-age indices were lower among children who were at the most 12 years than those in the age range 13-7 years. The high level of poor nutritional status found among CLHIV in our study is consistent with the outcomes of a study conducted in Burkina Faso, which reported a high prevalence of malnutrition among HIV-infected children when compared with HIV-uninfected children (Poda et al., 2017).

About three-fifths of children who lost their parents to HIV/AIDS were found to be stunted compared with one in five children who had lost either mother or father to HIV/AIDS. A similar pattern was observed in terms of thinness and underweight. Notably, the differences in nutritional outcomes were more pronounced across the age, parental level of education, and survival status of the children's parents. These poor nutritional outcomes may negatively affect the health and well-being of children, especially those among CLHIV.

Providing appropriate support and care for OVC poses a formidable challenge for both families and the society at large (Nsagha, 2012). In the context of ART, there is a dire need for stakeholders to include the provision of necessary diets for children (Ethiopia FMoH, 2008; National Agency for the Control of AIDS (NACA), 2015). This assumes significance because these vulnerable children have no access to important dietary intakes. While the increase in ART coverage among HIV-positive children is a welcome development and should indeed be sustained, effective interventions to ameliorate the poor nutritional status of these beleaguered children is paramount. Similar submissions have been made in the literature (Stover et al., 2015; Sunguya et al., 2011).

The deleterious effect of daily intake of antiretroviral drugs without eating a balanced diet can compromise the health of the children (Sunguya et al., 2011) besides negatively impacting the efficacy of the ART regimens. Sustainable maintenance of good nutrition could lead to a better response to treatment, reverse HIV progression, prolong health, and delay the progression of HIV to AIDS (Fekadu et al., 2015; Matara et al., 2015; Nsagha, 2012). In this context, several attempts have been made to break the linkage between HIV and malnutrition. The efforts include the model proposed by the Federal Ministry of Health, Ethiopia in 2008 (Ethiopia FMoH, 2008). The model hypothesized that giving adequate diets to CLHIV in order to meet their nutritional needs will lead to regaining or at least help maintain their weights, in which case macronutrient and micronutrient deficiencies would have been avoided. This, in turn, will lead to a strengthened immune system that is capable of warding off HIV and other infections, ultimately reducing the rate and duration of opportunistic infection and facilitating a probable reduction in the pace of progression to AIDS. Such a model should be adequately implemented in Nigeria. There is a need to provide or at least augment adequate feeding practices among CLHIV in Nigeria, especially among the orphaned CLHIV given that they have a higher likelihood of confronting adverse nutritional outcomes.

## 5 Conclusion

HIV-positive children in Oyo state, south-west Nigeria face a high burden of malnutrition, as indicated by the high prevalence of poor nutritional status among HIV-positive children undertaking daily ART regimens compared with the general population. For ART to be effective, there is the need for a strong support system both at home and at facility levels to ensure that the regimens are taken correctly and consistently. However, in addition to efficient ART, interventions to enhance the nutritional status of children is also essential. Such interventions should be targeted at the promotion as well as provision of adequate quality feeding patterns. An adequate balanced diet, proper hygiene, food safety, and nutritional management of any symptoms or identified malnutrition are inexorable interventions in order to minimize infection and eliminate malnutrition.

## Declarations

### Author contribution statement

A. Fagbamigbe: conceived and designed the experiments; performed the experiments; analyzed and interpreted the data; contributed reagents, materials, analysis tools or data; wrote the paper.

A. Adebowale, I. Ajayi: performed the experiments; contributed reagents, materials, analysis tools or data; wrote the paper.

### Funding statement

The authors received a seed grant for data collection from the Medical Education Partnership Initiative in Nigeria (MEPIN) Mentored Research Award been part of an NIH grant-supported research funded by the Fogarty International Centre, the office of AIDS Research and the National Human Genome Research Institute of the National Institute of Health, the Health Resources and Services Administration (HRSA) and the Office of the U.S. Global AIDS Coordinator under Award Number R24TW008878.

### Competing interest statement

The authors declare no conflict of interest.

### Additional information

No additional information is available for this paper.
